# Regulation of Diet-Induced Adipose Tissue and Systemic Inflammation by Salicylates and Pioglitazone

**DOI:** 10.1371/journal.pone.0082847

**Published:** 2013-12-23

**Authors:** Myung-Sunny Kim, Yasuhiko Yamamoto, Kyungjin Kim, Nozomu Kamei, Takeshi Shimada, Libin Liu, Kristin Moore, Ju Rang Woo, Steven E. Shoelson, Jongsoon Lee

**Affiliations:** 1 The Joslin Diabetes Center and Department of Medicine, Harvard Medical School, Boston, Massachusetts, United States of America; 2 Korea Food Research Institute, Seongnam, Republic of Korea; 3 Kanazawa University Graduate School of Medical Sciences, Kanazawa, Japan; University of Bari, Italy

## Abstract

It is increasingly accepted that chronic inflammation participates in obesity-induced insulin resistance and type 2 diabetes (T2D). Salicylates and thiazolidinediones (TZDs) both have anti-inflammatory and anti-hyperglycemic properties. The present study compared the effects of these drugs on obesity-induced inflammation in adipose tissue (AT) and AT macrophages (ATMs), as well as the metabolic and immunological phenotypes of the animal models. Both drugs improved high fat diet (HFD)-induced insulin resistance. However, salicylates did not affect AT and ATM inflammation, whereas Pioglitazone improved these parameters. Interestingly, HFD and the drug treatments all modulated systemic inflammation as assessed by changes in circulating immune cell numbers and activation states. HFD increased the numbers of circulating white blood cells, neutrophils, and a pro-inflammatory monocyte subpopulation (Ly6C^hi^), whereas salicylates and Pioglitazone normalized these cell numbers. The drug treatments also decreased circulating lymphocyte numbers. These data suggest that obesity induces systemic inflammation by regulating circulating immune cell phenotypes and that anti-diabetic interventions suppress systemic inflammation by normalizing circulating immune phenotypes.

## Introduction

Sub-acute inflammation may play important roles in the development of obesity-induced insulin resistance [1]–[4]. Adipose tissue (AT) macrophages (ATMs) are believed to play a key role in this process [5], [6]. This is supported by observations that obesity increases ATM numbers in humans and animal models; anti-diabetic interventions decrease ATM numbers; and the ablation of various genes in myeloid cells regulates the development of obesity-induced AT inflammation and systemic insulin resistance. Recent studies looking at ATMs have focused largely on the immunophenotypes that are changed by obesity, and in particular M1 and M2 phenotypes [7]. M1 macrophages mediate pro-inflammatory responses by producing pro-inflammatory cytokines, such as IL-6 or TNF-α, whereas M2 macrophages express unique signature molecules, including anti-inflammatory genes (IL-10 and TGF-β) and tissue repair/remodeling genes. M1 macrophages in AT are often defined as being CD11c^+^, and numerous studies have shown that obesity increases CD11c^+^ ATM numbers [7], [8]. Hence, it is generally thought that obesity shifts the ATM balance from M2 to a more M1 phenotype, and that anti-diabetic interventions, such as thiazolidinediones (TZDs), reverse this shift along with decreasing CD11c^+^ ATM numbers.

NFκB is an important regulator of inflammation. We have previously shown that obesity activates the IKKβ/NFκB pathway in animals and that inhibition of this pathway by salicylates improves obesity-induced insulin resistance [9], [10]. Subsequently, it was shown that systemic or myeloid cell-specific deletion of IKKβ or upstream molecules of the IKKβ/NFκB pathway, including TLR4, protects mice from the development of obesity-induced insulin resistance [9], [11]. These data show that NFκB plays a critical role in the development of obesity-induced inflammation and insulin resistance in murine models. We also found that salsalate improves glycemic control in patients with impaired glucose tolerance (IGT) or T2D and that this correlated with the inhibition of NFκB in circulating leukocytes [12], [13]. However, it has not been studied yet how salicylates affect AT and ATM inflammation to improve insulin sensitivity. This question was addressed in the present study.

## Materials and Methods

### Animals

All animal experiments were conducted in the Joslin Diabetes Center in accordance with the NIH guidelines under protocols approved by the Institutional Animal Care and Use Committee of the Joslin Diabetes Center. The mice were purchased from the Jackson Lab and maintained under a standard light cycle (12 hr light/dark) and allowed free access to water and food. They were then fed normal chow (NC, 10% fat, D12450B, Research Diets, Inc.) or a high fat diet (HFD, 60% fat, D12492, Research Diets, Inc.) to induce insulin resistance. Salicylates (Sal, 3 g/kg diet) or Pioglitazone (Pio, 100 mg/kg diet) was incorporated into a HFD and administered for the indicated time. Metabolic phenotypes were measured after overnight fasting. Insulin resistance index, HOMA-IR, was calculated based on fasting glucose and insulin levels [14].

### Histology

After adipose tissues were fixed with formalin and imbedded in paraffin, 4 µm thick tissue sections were prepared at the Joslin’s Histology core. The slides were then stained with hematoxylin and eosin and examined under an Olympus light microscope.

### Flow Cytometric Analyses

Stromal vascular cells (SVCs) were isolated from epididymal AT by using a well-established collagenase method [15]. RBCs were lysed with ACK lysis buffer (Biowhittaker), after which the remaining cells were stained with fluorophore-conjugated antibodies specific for cell surface markers (Table S1). ATMs were isolated as described (Figure S1). With regard to cells from the blood, blood was collected from the tail vein in the presence of 5 mM EDTA, incubated with BD Fc Block, stained and lysed with FACS Lysing Solution (BD Biosciences). The stained cells were then analyzed by LSRII and the data were further analyzed by using the FlowJo software. Complete blood counts (CBCs) were measured by using a Hemavet 950 (Drew Scientific Inc.).

### Real-time RT-PCR

Total RNA from AT and ATMs was prepared by using an RNeasy Lipid Kit (Qiagen). RNA from ATMs was further amplified with a MessageAmpII aRNA kit (Ambion). Thereafter, cDNA was generated by using an Advantage RT-PCR kit (ClonTech). Gene expression levels were determined by real-time RT-PCR. The primer sequences and probes that were used are listed in Tables S2 and S3.

### Microarray Analysis

ATMs were sorted by FACS and their total RNA was isolated by Trizol (Invitrogen) and amplified twice by using a MessageAmpII aRNA kit (Ambion). The cRNA was then labeled with biotin by using a MessageAmpII Biotin Enhanced aRNA kit (Ambion). The resulting cRNAs were hybridized to M430 2.0 chips (Affymetrix) and the chips were scanned at the Joslin’s Genomics Core. Thereafter, the data were processed, normalized, analyzed, and visualized by GenePattern software modules.

### Statistical Analysis

The results were expressed as means ± S.E.M. The data were analyzed by using unpaired *t*-tests. Differences were considered to be statistically significant at p<0.05.

## Results

### Salicylates and Pioglitazone Improve Diet-induced Insulin Resistance

**Figure 1:**
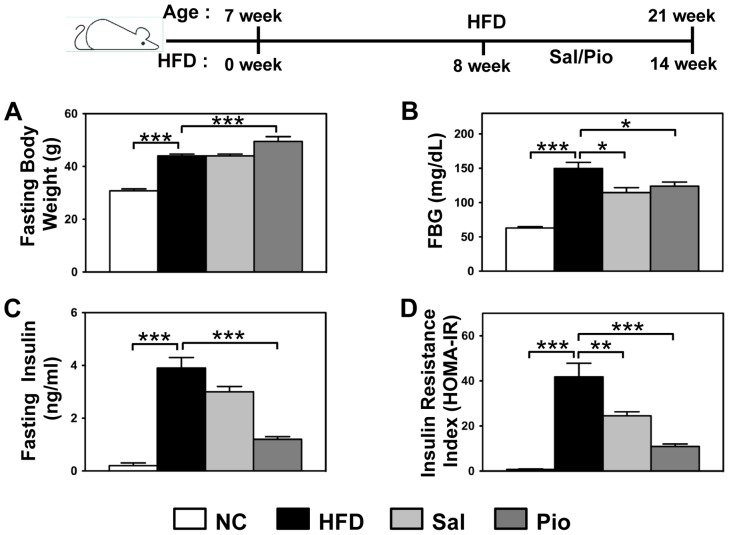
Treatment with Pio and/or Sal improves HFD-induced insulin resistance. C57BL/6 male mice (n = 8 per group) were given a HFD starting at 7 weeks of age. After 8 weeks on HFD, the animals were fed for another 6 weeks with HFD alone or HFD plus salicylates (Sal, 3 g/kg diet) or Pioglitazone (Pio, 100 mg/kg diet). The mice fed NC served as a control. After overnight fasting, the metabolic phenotypes were measured. The fasting body weight (A), fasting blood glucose levels (B), fasting serum insulin levels (C), and the insulin resistance index as HOMA-IR (D) are shown. *p<0.05; **p<0.01; ***p<0.001.

We first compared the improvements of metabolic phenotypes in diet-induced insulin resistance by salicylates and Pioglitazone. Mice were fed a HFD for 8 weeks and then treated with salicylates (3 g/kg diet) or Pioglitazone (100 mg/kg diet) for another 6 weeks. Their metabolic phenotypes were then measured (Figs. 1A–D). HFD increased the fasting body weight (FBW) by 43% and Pioglitazone increased it further by ∼13%. However, the salicylate treatments did not significantly change FBW, as compared to the HFD group (Fig. 1A). HFD also increased the fasting blood glucose (FBG) and fasting insulin levels (Fig. 1B & C), and therefore insulin resistance as determined by the HOMA-IR insulin resistance index (Fig. 1D). Both the salicylates and Pioglitazone treatments improved insulin sensitivity by lowering fasting blood glucose and insulin levels and therefore insulin resistance index (Figs. 1B–D), although the improvements in insulin sensitivity by Pioglitazone treatments were more profound than by salicylate treatments.

### Salicylates and Pioglitazone Regulate AT Inflammation Differently

**Figure 2:**
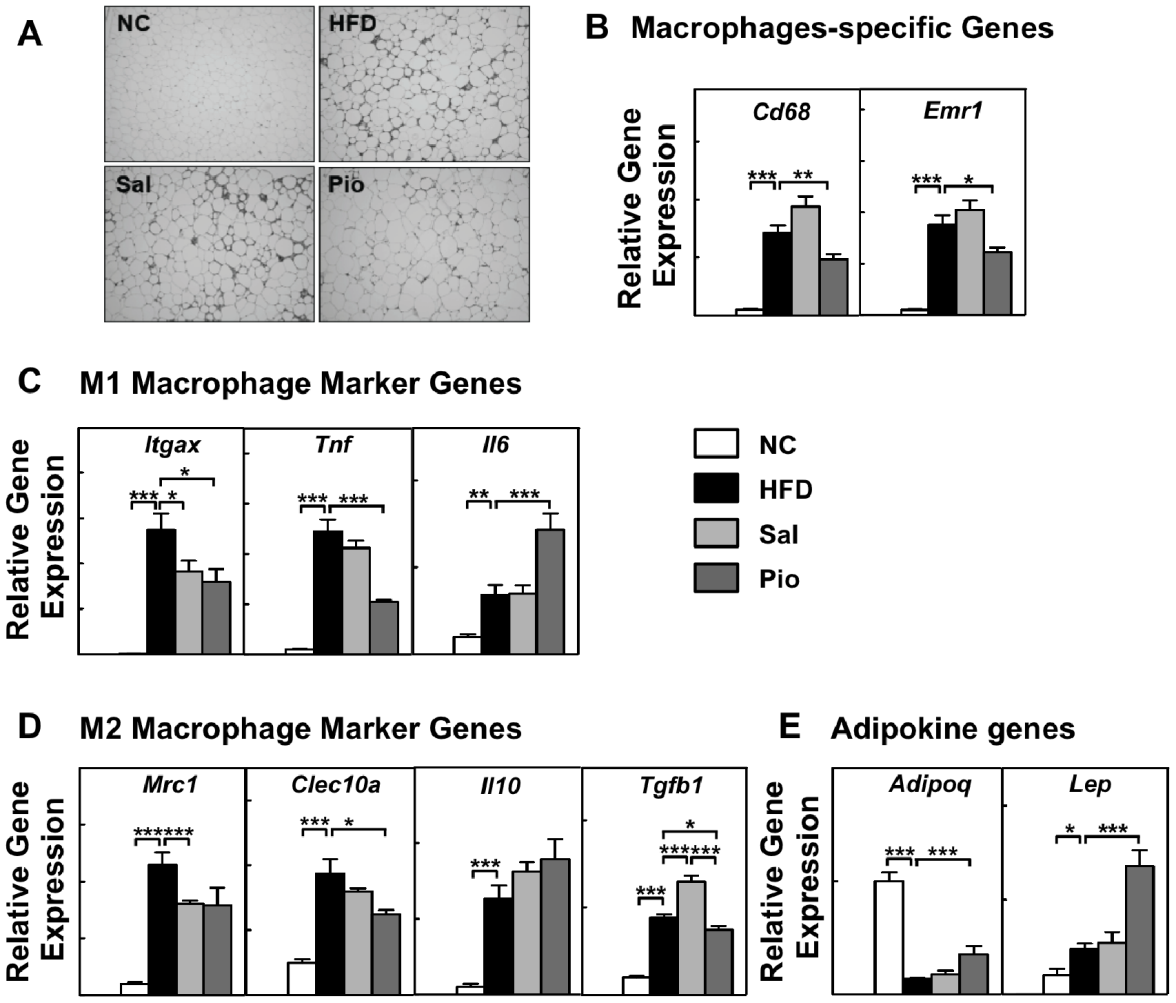
Sal and Pio treatment regulate inflammatory mediator gene expression in total AT differently. Total epididymal AT samples were obtained from the treatment study mice described in Figure 1 (n = 6–8 mice per group). AT inflammation was determined by assessing immune cell infiltration with H&E staining (A) and by examining the expression levels of inflammatory mediator genes by using real-time RT-PCR. The genes that were tested were macrophage-specific marker genes (B), M1 macrophage marker genes (C), M2 macrophage marker genes (D) and adipokine genes (E). The expression of the housekeeping gene GAPDH was used to normalize gene expression. *p<0.05; **p<0.01; ***p<0.001.

Recent studies strongly suggest that obesity and anti-diabetic interventions, including TZDs, regulate AT inflammation by modulating ATM phenotypes. Thus, the ability of salicylates to alter AT inflammation by this mechanism was examined. Hematoxylin and eosin staining of AT showed that HFD increased immune cell infiltration, and that Pioglitazone decreased this (Fig. 2A). In contrast, salicylate treatment was associated with the same degree of immune cell infiltration as HFD on its own.

Real-time RT-PCR of the total AT for the macrophage-specific marker genes *Cd68* and *Emr1* (F4/80) also indicated that HFD increased ATM infiltration (Fig. 2B). Consistent with the histology data (Fig. 2A), salicylate treatment did not change the HFD-induced gene expression of *Cd68* and *Emr1*. In contrast, Pioglitazone decreased it by ∼30%, which suggests that the latter treatment decreased obesity-induced ATM infiltration. HFD also increased the expression of M1 macrophage marker genes, including *Itgax* (CD11c), *Tnf* (TNFα) and *Il6* (Fig. 2C), but also M2 macrophage marker genes, including *Mrc1*, *Clec10a* (Mgl1), *Il10*, and *Tgfb1* (TGF-β) (Fig. 2D). Although salicylates did not alter the HFD-induced expression of the ATM marker genes (which reflects its lack of effect on ATM numbers) (Fig. 2B), it lowered *Itgax* and *Mrc1* gene expression and increased *Tgfb1* gene expression. In contrast, Pioglitazone decreased the expression levels of most M1 (pro-inflammatory) and M2 (anti-inflammatory) genes. The two exceptions were *Il6* and *Il10*: the gene expression of both interleukins tended to be increased by Pioglitazone. These data clearly showed that neither HFD nor the drug treatments caused the AT to adopt an exclusively pro- or anti-inflammatory status, or to shift wholly towards an M1 or M2 phenotype.

Since salicylate treatment increases circulating adiponectin levels in humans [13], the AT *Adipoq* (adiponectin) gene expression levels in the HFD-fed and treated mice were also measured. HFD significantly decreased *Adipoq* levels by 87% compared to the NC group. Salicylate treatment did not change this, whereas Pioglitazone slightly but significantly increased it (Fig. 2E). HFD also increased *Lep* (leptin) gene expression, whereas salicylate treatments did not alter it. However, Pioglitazone dramatically increased *Lep* expression levels by 2.9-fold compared to HFD (Fig. 2E). This is consistent with the ability of Pioglitazone to increase the fat weight (data not shown) and adipocyte size (Fig. 2A).

### Pioglitazone, but not Salicylates, Decreases ATM Infiltration and Activation

**Figure 3:**
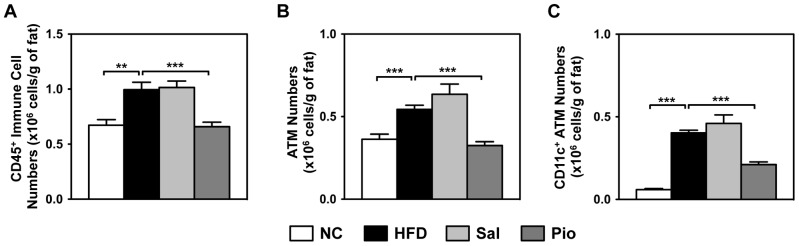
Treatment with Pio, but not Sal, decreases ATM numbers, as determined by flow cytometric analyses. Epididymal AT samples were prepared from the treatment study mice described in Figure 1 (n = 6–8 mice per group). The SVCs from the ATs of NC, HFD, Sal and Pio mice were isolated, stained with antibodies against cell- and activation-specific surface markers, and analyzed by flow cytometry by using LSRII and FlowJo software. ATMs were defined as CD45^+^, NK1.1^−^, CD49b^−^, CD90^−^, B220^−^, Gr-1^lo^, TER119^−^, CD11b^+^, and F4/80^+^. The cell numbers were normalized by AT weight to compensate for AT weight differences between groups. The normalized immune cell (CD45^+^) numbers (A), ATM numbers (B), and CD11c^+^ ATM numbers (C) are shown. *p<0.05; **p<0.01; ***p<0.001.

The AT gene expression data suggested that salicylates do not alter AT inflammation, including ATM infiltration. To assess this further, the changes in ATM numbers and their activation were quantitated by flow cytometric analysis (Fig. S1). When the numbers of total AT immune cells (defined as CD45^+^) were quantitated, HFD increased these cell numbers by 48% compared to the NC group (Fig. 3A). Although salicylates did not change these AT immune cell numbers, Pioglitazone caused them to drop by 51% compared to the HFD group. Similar to the changes in total AT immune cell numbers, HFD increased the numbers of ATMs (defined as CD45^+^, NK1.1^−^, CD49b^−^, CD90^−^, B220^−^, Gr-1^lo^, TER119^−^, CD11b^+^, and F4/80^+^, see Fig. S1) by 50% and Pioglitazone normalized them to the level of the NC control group (Fig. 3B). However, consistent with the *CD68* and *Emr1* gene expression data (Fig. 2B), salicylates did not alter ATM numbers. HFD also increased the number of putative M1 ATMs (defined as CD11c^+^ ATMs) by 6.8-fold while Pioglitazone halved this number and salicylates had no effect (Fig. 3C). Thus, Figures 2 and 3 show clearly that salicylates do not alter HFD-induced ATM infiltration whereas Pioglitazone suppress it.

### Salicylates do not Alter HFD-induced ATM Gene Expression Changes

**Figure 4:**
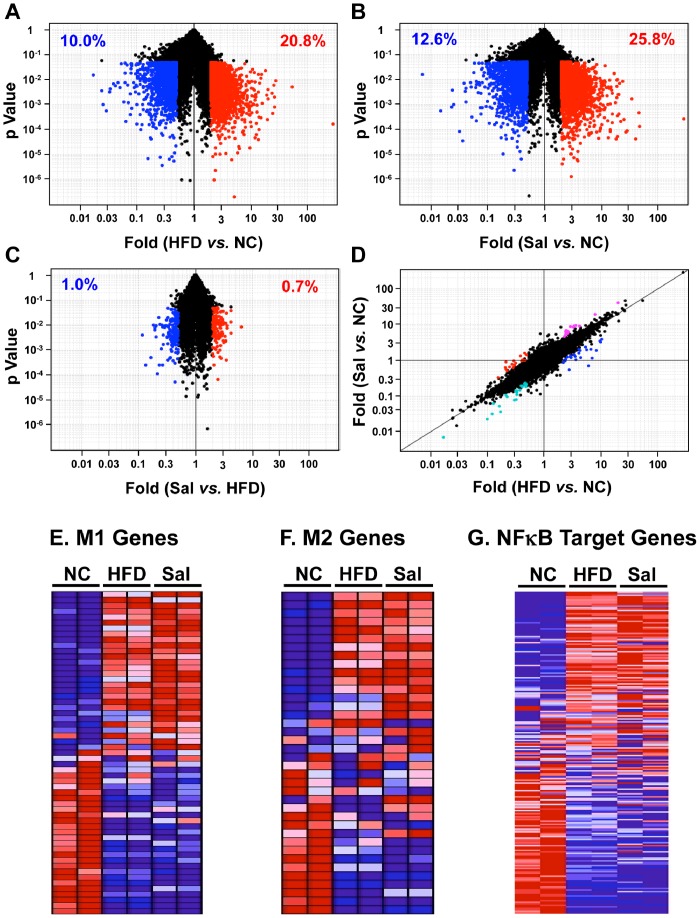
Microarray analysis of sorted ATMs from the NC, HFD and Sal groups. The ATMs were isolated from the ATs of the NC, HFD and Sal groups of the treatment study (n = 2 mice per group). Their RNAs were purified, amplified and analyzed by Affymetrix microarray analysis. The HFD *vs.* NC (A), Sal *vs.* NC (B), and Sal *vs.* HFD (C) gene expression profiles were compared by Volcano plots. Genes showing more than 2-fold differences between groups that were significant (p<0.05) are indicated as red dots if they were upregulated and as blue dots if they were downregulated genes. The genes in HFD and Sal mice that were differentially regulated relative to NC are shown in a FC/FC plot of (HFD *vs.* NC) *vs.* (Sal *vs.* NC) (D). The genes that showed a more than 2-fold difference in this comparison that was significant (p<0.05) are indicated as red dots (HFD down and Sal up), blue dots (HFD up and Sal down), cyan dots (HFD down and Sal further down), and pink dots (HFD up and Sal further up) (see Table 2). Heat maps show the differential gene expressions of M1 genes (E), M2 genes (F) and NFκB target genes (G).

Although salicylates did not change ATM infiltration, it is still possible that they can regulate ATM inflammation by altering ATM gene expression profiles. As it is not known which genes in ATMs are regulated by salicylates, an Affymetrix microarray technique was employed. The global gene expression profiles of FACS-sorted ATMs from NC, HFD, and HFD/salicylate-treated mice were first compared by using Volcano plots (Figs. 4A–C) and mean class expression plots (Fig. S2). The genes that were differentially expressed between the two populations (fold difference >2 and p<0.05) were shown as red dots if they were upregulated and as blue dots if they were downregulated. Compared to the NC group, 20.8% of the genes in the HFD group were upregulated and 10.0% were downregulated (Fig. 4A and Fig. S2A), and 25.8% of the genes in the Sal group were upregulated and 12.6% were downregulated (Fig. 4B and Fig. S2B). However, to our surprise, only a very small numbers of genes were significantly differently regulated between the HFD and Sal groups: only 0.7% of the genes in the Sal group were upregulated while 1.0% were downregulated (Fig. 4C and Fig. S2C). The top 10 genes that were differentially regulated in each two-group comparison are listed in Table 1. It is worth noting that only a few inflammatory mediator genes are on these lists.

**Table 1:** The top 10 genes that were differentially regulated in the HFD and Sal mouse groups.

*Upregulated in HFD mice relative to NC mice*						
Genes		NC	HFD			Sal
Matrix metallopeptidase 12 *(Mmp12)*		1.00±0.10	285.41±3.65			290.41±4.66
Ring finger protein 128 *(Rnf128)*		1.00±0.14	54.84±3.88			47.33±0.52
Glycoprotein (transmembrane) nmb *(Gpnmb)*		1.00±0.11	27.90±2.54			34.98±1.38
Interleukin 7 receptor *(Il7r)*		1.00±0.04	26.89±0.74			46.29±0.97
Glutaminyl-peptide cyclotransferase (glutaminyl cyclase) *(Qpct)*		1.00±0.29	26.43±1.09			24.24±2.09
Lipoprotein lipase *(Lpl)*		1.00±0.04	24.77±0.50			27.07±0.23
V-set and immunoglobulin domain containing 8 *(Vsig8)*		1.00±0.07	24.16±1.25			28.75±0.25
Fibroblast growth factor 13 *(Fgf13)*		1.00±0.29	20.66±0.77			12.73±0.46
Coagulation factor VII *(F7)*		1.00±0.10	20.29±1.21			41.00±0.24
Cathepsin K, *(Ctsk)*		1.00±0.04	20.17±0.30			19.93±0.93
***Upregulated in Sal mice relative to NC mice***						
**Genes**		**NC**	**HFD**			**Sal**
Matrix metallopeptidase 12 *(Mmp12)*		1.00±0.10	285.41±3.65			290.41±4.66
Ring finger protein 128 *(Rnf128)*		1.00±0.14	54.84±3.88			47.33±0.52
Interleukin 7 receptor *(Il7r)*		1.00±0.04	26.89±0.74			46.29±0.97
Coagulation factor VII *(F7)*		1.00±0.10	20.29±1.21			41.00±0.24
Glycoprotein (transmembrane) nmb *(Gpnmb)*		1.00±0.11	27.90±2.54			34.98±1.38
V-set and immunoglobulin domain containing 8 *(Vsig8)*		1.00±0.07	24.16±1.25			28.75±0.25
Lipoprotein lipase *(Lpl)*		1.00±0.04	24.77±0.50			27.07±0.23
Glutaminyl-peptide cyclotransferase (glutaminyl cyclase) *(Qpct)*		1.00±0.29	26.43±1.09			24.24±2.09
Potassium intermediate/small conductance calcium-activated channel, subfamily N, member 4 *(Kcnn4)*		1.00±0.03	16.11±0.50			22.18±0.30
Protocadherin 7 *(Pcdh7)*		1.00±0.07	18.41±2.66			21.32±1.68
***Upregulated in Sal mice relative to HFD mice***						
**Genes**		**NC**	**HFD**			**Sal**
immunoglobulin lambda chain, constant region 3 *(Igl-C3)*		1.16±0.05	1.00±0.04			6.45±0.49
Dedicator of cytokinesis 7 *(Dock7)*		0.82±0.29	1.00±0.29			4.23±0.56
Kinesin family member 13B *(Kif13b)*		0.86±0.05	1.00±0.02			4.16±0.36
Immunoglobulin lambda chain, constant region 2 *(Igl-C2)*		0.15±0.04	1.00±0.04			3.38±0.11
NFκB inhibitorξ *(Nfkbiz)*		0.96±0.01	1.00±0.09			3.04±0.03
Forkhead box P1 *(Foxp1)*		1.83±0.23	1.00±0.02			2.99±0.26
Eosinophil-associated, ribonuclease A family *(Ear)*		0.34±0.00	1.00±0.00			2.97±0.13
Lactate dehydrogenase C *(Ldhc)*		0.96±0.00		1.00±0.05		2.94±0.37
Acyl-Coenzyme A binding domain containing 3 *(Acbd3)*		1.50±0.04		1.00±0.03		2.90±0.14
Cut-like homeobox 1 *(Cux1)*		2.02±0.00		1.00±0.05		2.90±0.20
***Downregulated in HFD mice relative to NC mice***						
**Genes**	**NC**		**HFD**		**Sal**	
Aldo-keto reductase family 1, member B7 *(Akr1b7)*		1.00±0.12	0.02±0.00			0.01±0.00
Protamine 1 *(Prm1)*		1.00±0.06	0.02±0.00			0.02±0.00
Dysteine-rich secretory protein 1 *(Crisp1)*		1.00±0.02	0.03±0.00			0.02±0.00
Clusterin *(Clu)*		1.00±0.06	0.03±0.00			0.04±0.00
Carboxylesterase 3 *(Ces3)*		1.00±0.06	0.03±0.00			0.04±0.00
Complement factor D (adipsin) *(Cfd)*		1.00±0.08	0.04±0.00			0.04±0.00
Uroplakin 3B *(Upk3b)*		1.00±0.07	0.04±0.00			0.07±0.00
Uroplakin 1B *(Upk1b)*		1.00±0.07	0.05±0.00			0.05±0.00
Keratin 8 *(Krt8)*		1.00±0.20	0.05±0.00			0.06±0.00
Carbonic anhydrase 3 *(Car3)*		1.00±0.03	0.06±0.00			0.40±0.00
***Downregulated in Sal mice relative to NC mice***						
**Genes**	**NC**		**HFD**			**Sal**
Aldo-keto reductase family 1, member B7 *(Akr1b7)*		1.00±0.12	0.02±0.00			0.01±0.00
Cysteine-rich secretory protein 1 *(Crisp1)*		1.00±0.02	0.03±0.00			0.02±0.00
Selectin, platelet *(Selp)*		1.00±0.03	0.10±0.01			0.02±0.00
Protamine 1 *(Prm1)*		1.00±0.06	0.02±0.00			0.02±0.00
Serum amyloid A 3 *(Saa3)*		1.00±0.01	0.17±0.00			0.04±0.00
Complement factor D (adipsin) *(Cfd)*		1.00±0.08	0.04±0.00			0.04±0.00
Clusterin *(Clu)*		1.00±0.06	0.03±0.00			0.04±0.00
Carboxylesterase 3 *(Ces3)*		1.00±0.06	0.03±0.00			0.04±0.00
Uroplakin 1B *(Upk1b)*		1.00±0.07	0.05±0.00			0.05±0.00
Histidine decarboxylase, *(Hdc)*		1.00±0.02	0.16±0.00			0.05±0.00
***Downregulated in Sal mice relative to HFD mice***						
**Genes**		**NC**	**HFD**			**Sal**
Igκ chain variable 28 *(Igk-V28)*		0.16±0.01	1.00±0.06			0.12±0.01
Nudix (nucleoside diphosphate linked moiety X)-type motif 21 *(Nudt21)*		0.16±0.15	1.00±0.01			0.13±0.01
Fascin homolog 1, actin bundling protein *(Fscn1)*		0.88±0.02	1.00±0.07			0.18±0.03
Cytoplasmic polyadenylation element binding protein 2, Cpeb2		1.34±0.21	1.00±0.06			0.19±0.01
CD5 antigen-like *(Cd5l)*		0.51±0.02	1.00±0.04			0.20±0.02
GC-rich promoter binding protein 1 *(Gpbp1)*		1.68±0.29	1.00±0.06			0.20±0.05
Serum amyloid A 3 *(Saa3)*		5.84±0.03	1.00±0.00			0.21±0.01
Nudix (nucleoside diphosphate linked moiety X)-type motif 21 *(Nudt21)*		0.60±0.07	1.00±0.11			0.22±0.00
Selectin, platelet *(Selp)*		10.06±0.27	1.00±0.08			0.23±0.02
Selenoprotein W, muscle 1 *(Sepw1)*		3.07±0.47	1.00±0.02			0.25±0.00

Thereafter, the gene expression changes in HFD/NC vs. Sal/NC were detected by using a Fold Change *vs*. Fold Change (FC/FC) plot (Fig. 4D). In FC/FC plots, if genes of two populations (here, HFD or Sal) are similarly regulated compared to the control group (here, NC), most of the genes will be aligned along a diagonal line. However, if they are differently regulated, the genes will be away from the line. Figure 4D showed clearly that most of the genes aligned well along the diagonal line. Only 53 genes were differentially regulated (fold differences >2.0 and p<0.05) and these are listed in Table 2. Again, they included very few inflammatory mediator genes. These data together (Figs. 2–>4) showed strongly that salicylates do not improve obesity-induced insulin resistance by modulating ATM recruitment or activation.

**Table 2:** The genes that were up- or down-regulated in HFD and Sal mice relative to NC mice.

*Genes that were upregulated in HFD and downregulated in Sal (red dots in * *Fig. 4D* *)*			
Genes	NC	HFD	Sal
Igκ constant C/V28 region *(Igk-C/Igk-V28)*	0.12±0.03	1.00±0.01	0.23±0.00
Ubiquitin carboxy-terminal hydrolase L1 *(Uchl1)*	0.23±0.01	1.00±0.03	0.27±0.03
Ig joining chain *(Igj)*	0.15±0.02	1.00±0.08	0.31±0.02
Ser/Cys peptidase inhibitor *(Serpinb9b)*	0.10±0.02	1.00±0.03	0.32±0.01
Matrix metallopeptidase 3 *(Mmp3)*	0.10±0.00	1.00±0.00	0.35±0.01
Lysosomal acid lipase A *(Lipa)*	0.18±0.02	1.00±0.07	0.36±0.01
PRP19/PSO4 mammalian homolog *(Prpf19)*	0.28±0.01	1.00±0.03	0.36±0.01
Alpha fetoprotein *(Afp)*	0.10±0.01	1.00±0.06	0.38±0.05
Rock1 *(Rock1)*	0.46±0.04	1.00±0.08	0.40±0.01
Thymus cell antigen 1ξ *(Thy1)*	0.33±0.01	1.00±0.03	0.40±0.03
Isochorismatase domain containing 1 *(Isoc1)*	0.43±0.05	1.00±0.04	0.41±0.01
Interferonγ *(Ifng)*	0.29±0.03	1.00±0.09	0.42±0.03
Histone cluster 1H2a *(Hist1h2a)*	0.34±0.06	1.00±0.03	0.43±0.01
Arylacetamide deacetylase-like 1 *(Aadacl1)*	0.19±0.01	1.00±0.02	0.44±0.02
Glia maturation factorβ *(Gmfb)*	0.44±0.06	1.00±0.09	0.44±0.04
BMP-binding endothelial regulator *(Bmper)*	0.42±0.01	1.00±0.03	0.44±0.03
Vesicle-associated membrane protein B and C *(Vapb)*	0.34±0.00	1.00±0.05	0.46±0.00
ADP-ribosylation factor-like 6 interacting protein 5 *(Arl6ip5)*	0.39±0.06	1.00±0.00	0.48±0.03
IGF-2 mRNA binding protein 2 *(Igf2bp2)*	0.10±0.01	1.00±0.03	0.10±0.01
***Genes that were downregulated in HFD and upregulated in Sal (blue dots in *** ***Fig. 4D*** ***)***			
**Genes**	**NC**	**HFD**	**Sal**
Cut-like homeobox 1 *(Cux1)*	2.02±0.00	1.00±0.05	2.90±0.20
Stat 3 *(Stat3)*	4.71±0.05	1.00±0.04	2.70±0.00
Nischarin *(Nisch)*	4.08±0.48	1.00±0.07	2.66±0.06
RAB43 *(Rab43)*	2.37±0.02	1.00±0.00	2.48±0.18
Metastasis suppressor 1 *(Mtss1)*	4.47±0.65	1.00±0.02	2.27±0.04

### HFD does not Categorically Affect M1, M2, and NFκB Target Gene Clusters

Microarray dataset was analyzed further to determine molecular differences of the ATMs from the HFD and NC groups. The current paradigm in the field is that obesity polarizes the ATMs from M2 macrophages into M1 macrophages. However, the heat map for the M1 gene cluster showed clearly that HFD did not exclusively and categorically upregulate the expressions of the M1 cluster genes (Fig. 4E and Table S4A): while one-third of the M1 cluster genes were indeed upregulated by HFD (e.g. *Il7r* and *Itgax*), almost equal numbers of genes were not changed by HFD (e.g. *Il12a* and *Ccl2*) or even downregulated (e.g. *Il6* and *Ptgs2*). The M2 and NFκB target genes clusters also showed very similar patterns (Figs. 4F, 4G, and Tables S4B and S4C). Consistent with the previous results, salicylates did not significantly affect the expression of these genes. These data demonstrate clearly that ATMs cannot be exclusively categorized as either M1 or M2, as has been suggested previously. They also suggest strongly that with regard to the development of obesity-induced inflammation, individual inflammatory mediator genes must be studied instead of seeking to categorize ATMs into M1 or M2 phenotypes.

### Salicylates and Pioglitazone Regulate the M1 and M2 Gene Expression of ATMs Differently

**Figure 5:**
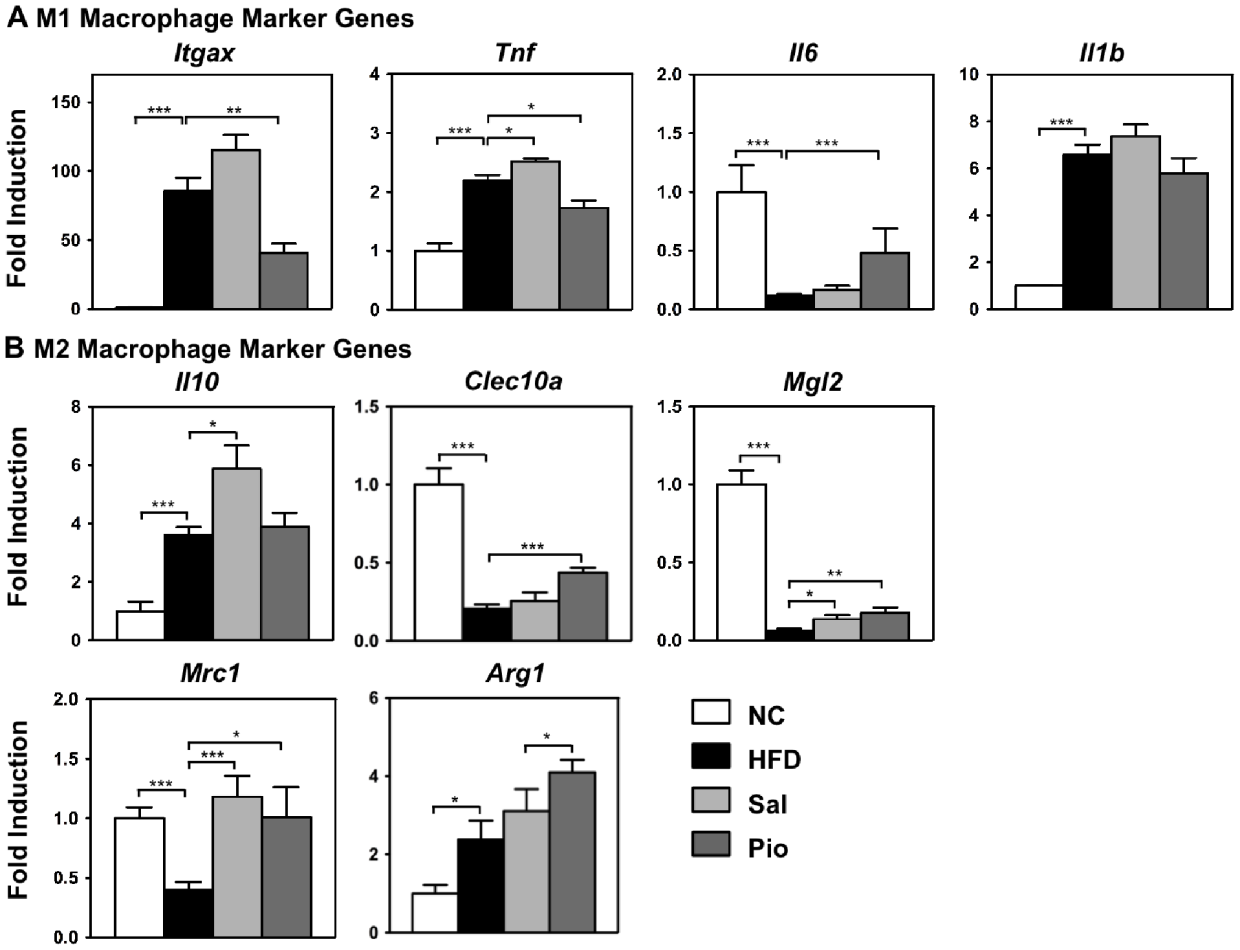
Pio and Sal regulate the M1 and M2 gene expressions of sorted ATMs differently. ATMs were sorted from the ATs of the NC, HFD, Sal and Pio groups in the treatment study described in Figure 1 (n = 6–8 mice per group). Their RNAs were then purified, amplified and analyzed by real-time RT-PCR. The genes that were tested were M1 (A) and M2 (B) genes. The housekeeping gene GAPDH was used to normalize gene expression. *p<0.05; **p<0.01; ***p<0.001.

Next, the M1 and M2 gene expressions of the ATMs in the microarray data were verified by using real-time RT-PCR and compared to the expression of M1 and M2 genes in the Pio ATMs (Fig. 5). Consistent with the microarray data, not all M1 macrophage marker genes were upregulated by HFD, nor were all M2 macrophage marker genes downregulated by HFD. For example, HFD increased *Tnf* gene expression but decreased *Il6* gene expression. Moreover, HFD downregulated *Clec10a* (Mgl1), *Mgl2* and *Mrc1* expression while upregulating *Il10* and *Arg1* gene expression. These data confirm the notion that HFD does not exclusively polarize ATMs into M1 or M2 phenotypes. Salicylates and Pioglitazone also regulated the expression of these genes in a manner that was inconsistent with the M1/M2 paradigm. For example, while Pioglitazone decreased the effect of HFD on the gene expression of *Itgax* and *Tnf* and increased *Il6* gene expression, it also increased the HFD-elevated *Arg1* gene expression further. Moreover, salicylates increased the expression levels of the pro-inflammatory *Itgax* and *Tnf* genes as well as the anti-inflammatory *Il10* gene. These data confirm that HFD and anti-diabetic interventions regulate inflammatory mediator genes in an individual and independent manner rather than as M1 or M2 clusters.

### HFD Induces Systemic Inflammation by Regulating Circulating Leukocytes, and Salicylates and Pioglitazone Normalize this

**Figure 6:**
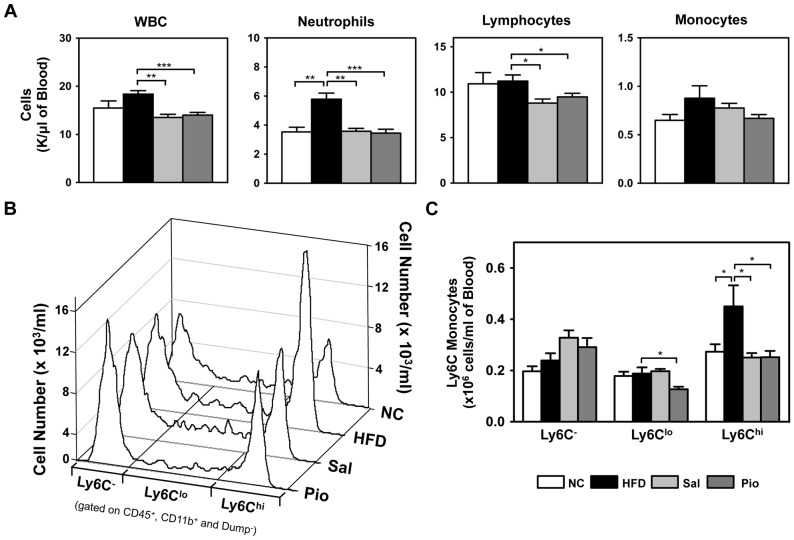
Obesity induces systemic inflammation by increasing the numbers of circulating immune cells and a pro-inflammatory monocyte subset, and Sal and Pio treatment reverse these increases. The CBCs of the mice from the treatment study described in Figure 1 were determined by using a Hemavet hematology analyzer (A). The circulating Ly6C^-^, Ly6C^lo^, and Ly6C^hi^ monocyte subpopulations were analyzed by flow cytometric analysis (B and C). *p<0.05; **p<0.01; ***p<0.001.

This study showed that salicylates did not improve insulin sensitivity by targeting the AT or ATMs. However, it was observed that the salicylates strongly regulated circulating immune cells. Analysis of CBCs revealed that HFD increased circulating WBC and neutrophil numbers and that salicylates and Pioglitazone normalized these numbers to the numbers seen in the NC group (Fig. 6A). The drug treatments also lowered circulating lymphocyte numbers, although HFD did not change them. We further examined the effect of obesity and the drug treatments on the changes in the lymphocyte subtype numbers in the circulation by using flow cytometry analyses (Fig. S3). We found that obesity and the anti-diabetic drugs changed the circulating lymphocyte subsets: HFD increased the numbers of CD4 T cells and B cells and Sal and Pio treatments reduced the numbers of CD4 and CD8 T and B cells, although these changes did not achieve statistical significance.

None of the treatments changed the circulating monocyte numbers (Fig. 6A). However, it has been well documented that monocyte subpopulations that are isolated on the basis of their Ly6C expression play an important role in regulating inflammation in various disease models [16]. The Ly6C^hi^ monocyte subpopulation expresses CCR2, moves into the local inflammatory sites in a MCP1-dependent manner, and then differentiates into macrophages that regulate inflammation. Hence, Ly6C^hi^ monocytes are often denoted as an inflammatory monocyte subset. In contrast, the Ly6C^-^ monocytes express CX_3_CR1 instead of CCR2 and are considered to be the residential monocytes. Flow cytometric analysis showed that HFD dramatically increased the size of the Ly6C^hi^ monocyte subpopulation without changing the numbers of the Ly6C^lo^ or Ly6C^-^ populations (Figs. 6B and C and Fig. S4). Sal and Pio treatments decreased the size of the Ly6C^hi^ population to the level of the NC group while marginally increasing the Ly6C^-^ subpopulation. Pioglitazone also significantly lowered the Ly6C^lo^ population number (Fig. 6C). These data show clearly that HFD treatment increased the numbers of circulating WBCs, neutrophils, and the Ly6C^hi^ pro-inflammatory monocytes, and that both anti-diabetic interventions normalized this systemic inflammation.

## Discussion

The main aim of this study was to examine the effect of salicylates on AT and ATM inflammation and to compare this with the effects of Pioglitazone. We used Pioglitazone as a control in this study because it has previously been shown to suppress obesity-induced AT inflammation, especially of ATMs. However, TZDs including Pioglitazone have additional effects on adipocyte differentiation and the redistribution of lipids from ectopic sites like the liver into AT, which improve insulin sensitivity and glycemic control. We were surprised by the lack of an effect of salicylates on either AT or ATM inflammation, under conditions where Pioglitazone decreased ATM numbers and suppressed inflammatory gene expression in ATMs. This was shown by the inflammatory mediator gene expression profiles of AT and sorted ATMs, the flow cytometric analyses of ATMs, and most conclusively, the microarray gene expression profile analyses. Thus, the present study suggests that the improvement of insulin sensitivity by salicylates does not require the suppression of HFD-induced AT or ATM inflammation.

These observations lead to the question: what is the target of salicylates for the suppression of HFD-induced inflammation? Because obesity, insulin resistance, and T2D are systemic diseases, we postulated that obesity-induced inflammation is also systemic, namely, that obesity changes the numbers and/or activations of the circulating (systemic) immune cells. Indeed, HFD significantly increased the numbers of circulating immune cells, including neutrophils. Total monocyte numbers were not changed by HFD or the treatments. However, it is now well established that Ly6C^hi^ monocytes play a pro-inflammatory role in various inflammatory diseases, including atherosclerosis [17], [18], and we have shown that HFD also increases the Ly6C^hi^ monocyte subpopulation. Furthermore, salicylates and Pioglitazone decreased the numbers of WBCs, neutrophils, lymphocytes and Ly6C^hi^ monocytes while concurrently improving insulin sensitivity.

Interestingly, our clinical study showed that salicylates halve the NFκB activity in circulating immune cells [12]. This phenomenon coincides with the salicylate-induced 50% decrease in Ly6C^hi^ monocyte numbers compared to the HFD group in the present study (Fig. 6C). Notably, Ly6C^hi^ monocytes express CCR2, which mediates the migration of monocytes into local inflammatory sites. However, the present study showed that salicylates, unlike Pioglitazone, did not decrease ATM infiltration in obesity. Consequently, the macrophage infiltration of the liver was assessed in the present study by measuring macrophage marker gene (*Cd68* and *Emr1*) expression by using real-time RT-PCR (Fig. S5). However, while Pioglitazone decreased HFD-induced macrophage infiltration into the liver, salicylates did not. This suggests that salicylates may not regulate monocyte migration into local tissues. How, then, do obesity and salicylates regulate systemic inflammation and insulin resistance? One possibility is that circulating activated immune cells produce inflammatory cytokines. It has been established that Ly6C^+^ monocytes can produce inflammatory cytokines [16], [19]. In addition, neutrophils are fully activated when they egress from the bone marrow and therefore can immediately produce inflammatory cytokines in the circulation in response to environmental insults such as obesity. Although the absolute amounts produced by these circulating cells may be less than that generated by tissue resident cells including ATMs or AT neutrophils, there are far greater numbers of circulating immune cells than local immune cells. Therefore, the total cytokine output of the circulating immune cells may be much greater than that of tissue resident leukocytes. Obesity and anti-diabetic drugs may impact inflammation and insulin resistance through systemic effects on circulating immune cell numbers and activation. To test this, we examined the circulating cytokine levels in the NC, HFD, Sal and Pio groups by using multiple assay methods, including the Luminex system. However, in our hands, many cytokines in the circulation, including IFNγ, IL-4 and IL-15, are under the limits of detection, and others (IL-1β, IL-6, TNFα and RANTES) did not differ regardless of diet or treatment (data not shown). It may be that because obesity-induced inflammation is low grade and subacute, activated immune cells may produce much lower levels of inflammatory mediators than the current systems (ELISA or Luminex system) can detect. Another possibility is that we did not select the right inflammatory mediators for this experiment because obesity-induced inflammation may differ markedly from the classical inflammation induced by infections and autoimmunity. There are more than 100 different inflammatory mediators (including cytokines) that play distinctive roles in response to the various inflammatory challenges. However, with the current assay systems, we could measure only limited numbers of cytokines that we have chosen based on the studies from the classical immunological studies. Therefore, it is possible that obesity and anti-diabetic treatments regulate different types of non-classical inflammatory mediators that we did not assess in this study.

Nevertheless, clinical studies support this and are consistent with a hypothesized role for circulating leukocytes in the pathogenesis of insulin resistance. Epidemiological studies have shown that high circulating neutrophil and WBC counts predict incident T2D and atherosclerosis, and poor outcomes after myocardial infarctions and stroke [20]–[26]. Moreover, the TINSAL-T2D clinical trial showed that salicylates lowers WBC, neutrophil and lymphocyte counts in patients with T2D concomitant with improvements in glycemic control [27]. Of the inflammatory variables tested in TINSAL-T2D, changes in circulating leukocyte counts were among the most robust. Clinical trials have also shown that Pioglitazone lowers circulating leukocyte counts along with improving glycemic control [28]–[30]. However, these earlier findings were largely ignored, perhaps because the counts remained well within normal ranges. Future studies are needed to assess potential roles of circulating leukocytes on insulin resistance as well as other cardiometabolic complications of obesity.

These findings together suggest strongly that salicylates improve insulin resistance and glycemic controls by regulating systemic inflammation. However, it is still possible that salicylate’s improvements of dysglycemia may be through multiple mechanisms, including mechanisms other than suppression of obesity-induced inflammation.

The microarray data in the present study not only confirmed conclusively that salicylates did not target ATMs, they also revealed several other interesting findings that may be underappreciated in the field of obesity research. First, these data suggest strongly that ATMs cannot be categorized according to the binary system of M1 and M2 phenotypes; instead, they should be categorized on the basis of individual inflammatory mediator genes (e.g. *Tnf vs*. *Il6*). Second, the changes in the expression of genes in the AT were much greater than those of sorted ATMs: for example, while sorted ATMs from HFD-fed mice expressed ∼2-fold higher levels of *Tnf* relative to NC mice, the total AT from these HFD-fed mice expressed ∼25-fold higher levels of *Tnf*. This is mainly due to the compounding effects of increased ATM numbers and their increased gene expression levels, and it may be one of the mechanisms by which obesity amplifies AT inflammation. Moreover, other immune cells may also contribute to the degree of AT inflammation; this may be responsible for the differential gene expression of sorted ATMs and total AT regarding cytokines such as *Il6*.

In summary, salicylate improved obesity-induced insulin resistance without affecting AT and ATM inflammation. However, it was also observed that obesity induced systemic inflammation by modulating the numbers of circulating WBCs, neutrophils, and the Ly6C^hi^ pro-inflammatory monocytes, and that salicylates and Pioglitazone suppressed this systemic inflammation, as shown by their ability to reverse the HFD-induced changes in the numbers of circulating immune cells and their subpopulations. Hence, these data may provide new insights into how HFD and anti-diabetic drugs regulate inflammation, namely, by modulating systemic inflammation.

## Supporting Information

Figure S1 Gating strategy for flow cytometric analysis of ATMs. After SVCs were isolated from AT by using a collagenase method, they were stained with antibodies against cell-specific markers and subjected to flow cytometric analysis with LSR II. The data were analyzed by FlowJo software with the following gating strategy. After the appropriate cells were selected by FSC/SSC gating, the aggregated cells were removed by FSC-W/SSC gating (A). The live cells were then selected by propidium iodide (PI)^-^ staining (B). Thereafter, the total leukocytes were selected as CD45^+^ (C). The lymphocytes and RBCs were excluded by removing the NK1.1^+^ (NK and NKT cells), CD49b^+^ (NK cells), B220^+^ (B cells), CD90^+^ (T cells), and TER119^+^ (RBC) cells (D). The neutrophils were removed by eliminating the Gr-1^hi^ cells (E). Finally, ATMs were determined by identifying the CD11b^+^ and F4/80^+^ cells (F). Hence, in this gating strategy, ATMs were defined as CD45^+^, NK1.1^−^, CD49b^−^, CD90^−^, B220^−^, Gr-1^lo^, TER119^−^, CD11b^+^ and F4/80^+^.
(TIF)

Figure S2 Mean Class Expression comparison of the microarray data in Figure 4. On the basis of the microarray data of Figure 4, the following mean class expression profile comparisons were performed: HFD *vs.* NC (A), Sal *vs.* NC (B), and Sal *vs.* HFD (C). Genes that showed a more than 2-fold difference between groups that was significant (p<0.05) were indicated as red dots if they were upregulated and as blue dots if they were downregulated.
(TIF)

Figure S3 Treatment with Pio and Sal decreases circulating T and B cells, as determined by flow cytometric analyses. Blood was collected from the tail vein in the presence of 5 mM EDTA, incubated with BD Fc Block, stained with antibodies and lysed with FACS Lysing Solution (BD Biosciences). The cells were then analyzed with LSR II and the data were analyzed by FlowJo software.
(TIF)

Figure S4 Gating strategy for flow cytometric analysis of circulating monocytes. Whole blood was incubated with FcBlock to block the non-specific binding of Abs and then stained with antibodies against cell-specific surface markers. After staining, the RBCs were removed by lysing with FACS Lysing Solution (BD Biosciences). The cells were then analyzed with LSR II. The data were analyzed by FlowJo software with the following gating strategy. Aggregated cells were first removed by FSC-W/SSC gating (A). The total leukocytes were identified by positivity for CD45 (B). The neutrophils were removed on the basis of their Gr-1^hi^ status (C). The lymphocytes and RBCs were excluded by removing the NK1.1^+^ (NK and NKT cells), CD49b^+^ (NK cells), B220^+^ (B cells), CD90^+^ (T cells), and TER119^+^ (RBC) cells (D). Monocytes were selected on the basis of their CD11b positivity (E). The monocyte subpopulations were determined on the basis of their Ly6C expression levels (F).
(TIF)

Figure S5 Pio, but not Sal, treatment decreased HFD-induced macrophage infiltration in the liver. Liver samples from the treatment study described in Figure 1 were prepared (n = 6–8 mice per group). mRNA was purified and the expression levels of macrophage-specific genes were determined by real-time RT-PCR. Gene expression was normalized by using the cyclophilin gene. *p<0.05; **p<0.01.
(TIF)

Table S1 Cell surface markers that were used for the flowcytometry analyses.
(TIF)

Table S2 TaqMan probes used in this study.
(TIF)

Table S3 Primer sequences used in this study.
(TIF)

Table S4 (ZIP)
