# Supplementary figures and images for: Regulation of Diet-Induced Adipose Tissue and Systemic Inflammation by Salicylates and Pioglitazone
Source: PLoS One. 2013 Dec 23;8(12):e82847. doi: 10.1371/journal.pone.0082847 (PMC3871540; doi:10.1371/journal.pone.0082847)

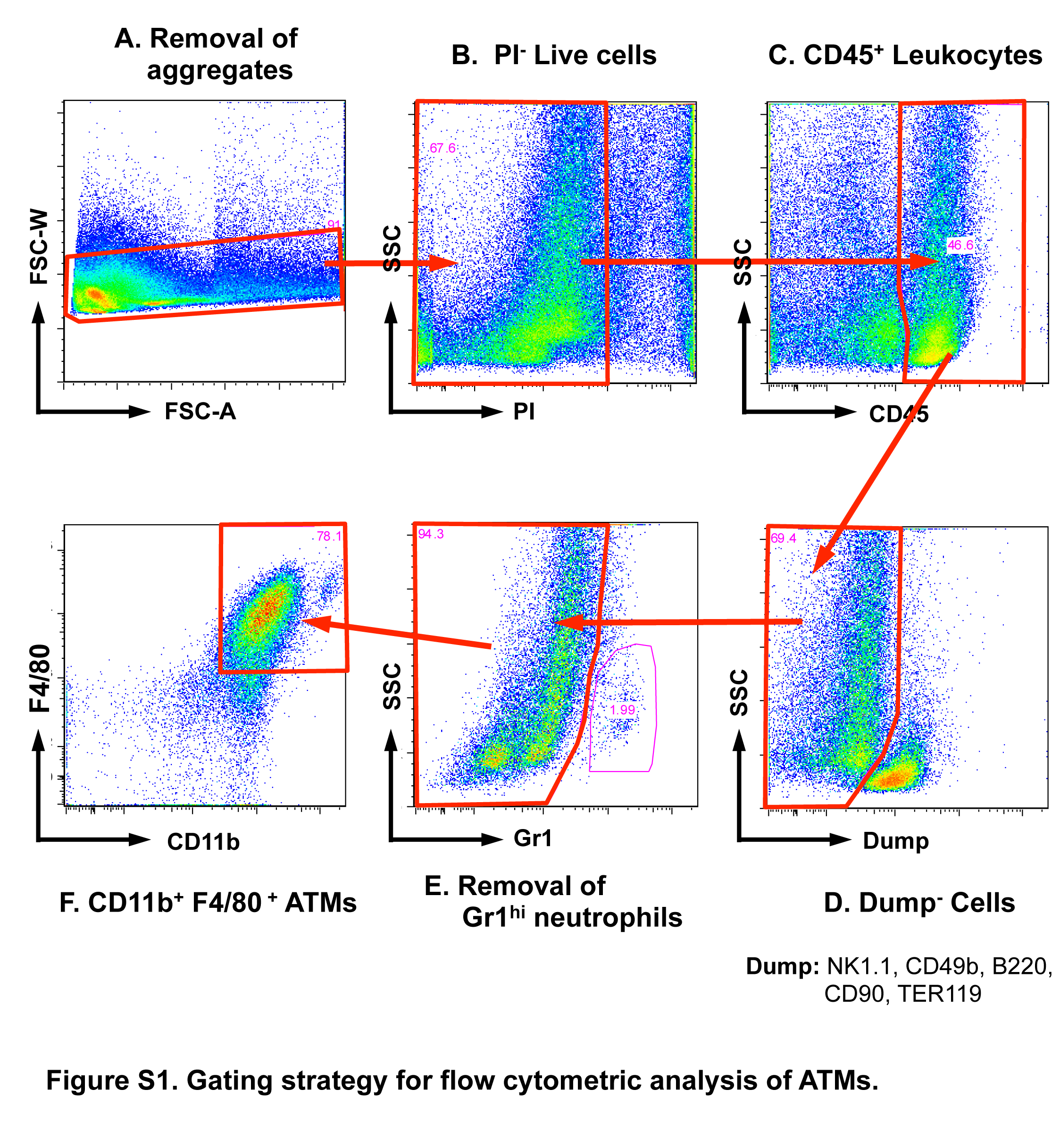

Supplement: Figure S1 — Gating strategy for flow cytometric analysis of ATMs. After SVCs were isolated from AT by using a collagenase method, they were stained with antibodies against cell-specific markers and subjected to flow cytometric analysis with LSR II. The data were analyzed by FlowJo software with the following gating strategy. After the appropriate cells were selected by FSC/SSC gating, the aggregated cells were removed by FSC-W/SSC gating (A). The live cells were then selected by propidium iodide (PI)- staining (B). Thereafter, the total leukocytes were selected as CD45+ (C). The lymphocytes and RBCs were excluded by removing the NK1.1+ (NK and NKT cells), CD49b+ (NK cells), B220+ (B cells), CD90+ (T cells), and TER119+ (RBC) cells (D). The neutrophils were removed by eliminating the Gr-1hi cells (E). Finally, ATMs were determined by identifying the CD11b+ and F4/80+ cells (F). Hence, in this gating strategy, ATMs were defined as CD45+, NK1.1−, CD49b−, CD90−, B220−, Gr-1lo, TER119−, CD11b+ and F4/80+. (TIF) [file pone.0082847.s001.tif]

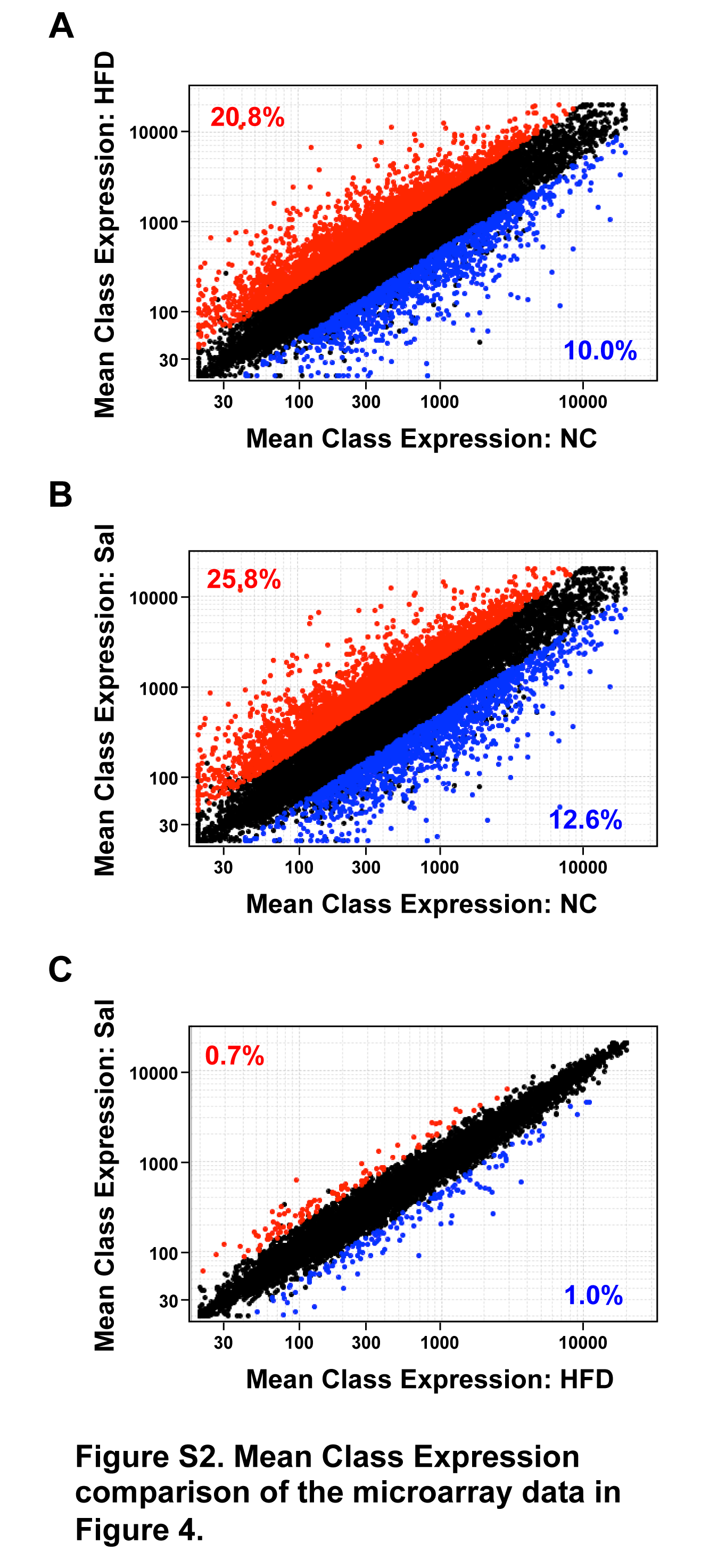

Supplement: Figure S2 — Mean Class Expression comparison of the microarray data in Figure 4. On the basis of the microarray data of Figure 4, the following mean class expression profile comparisons were performed: HFD vs. NC (A), Sal vs. NC (B), and Sal vs. HFD (C). Genes that showed a more than 2-fold difference between groups that was significant (p<0.05) were indicated as red dots if they were upregulated and as blue dots if they were downregulated. (TIF) [file pone.0082847.s002.tif]

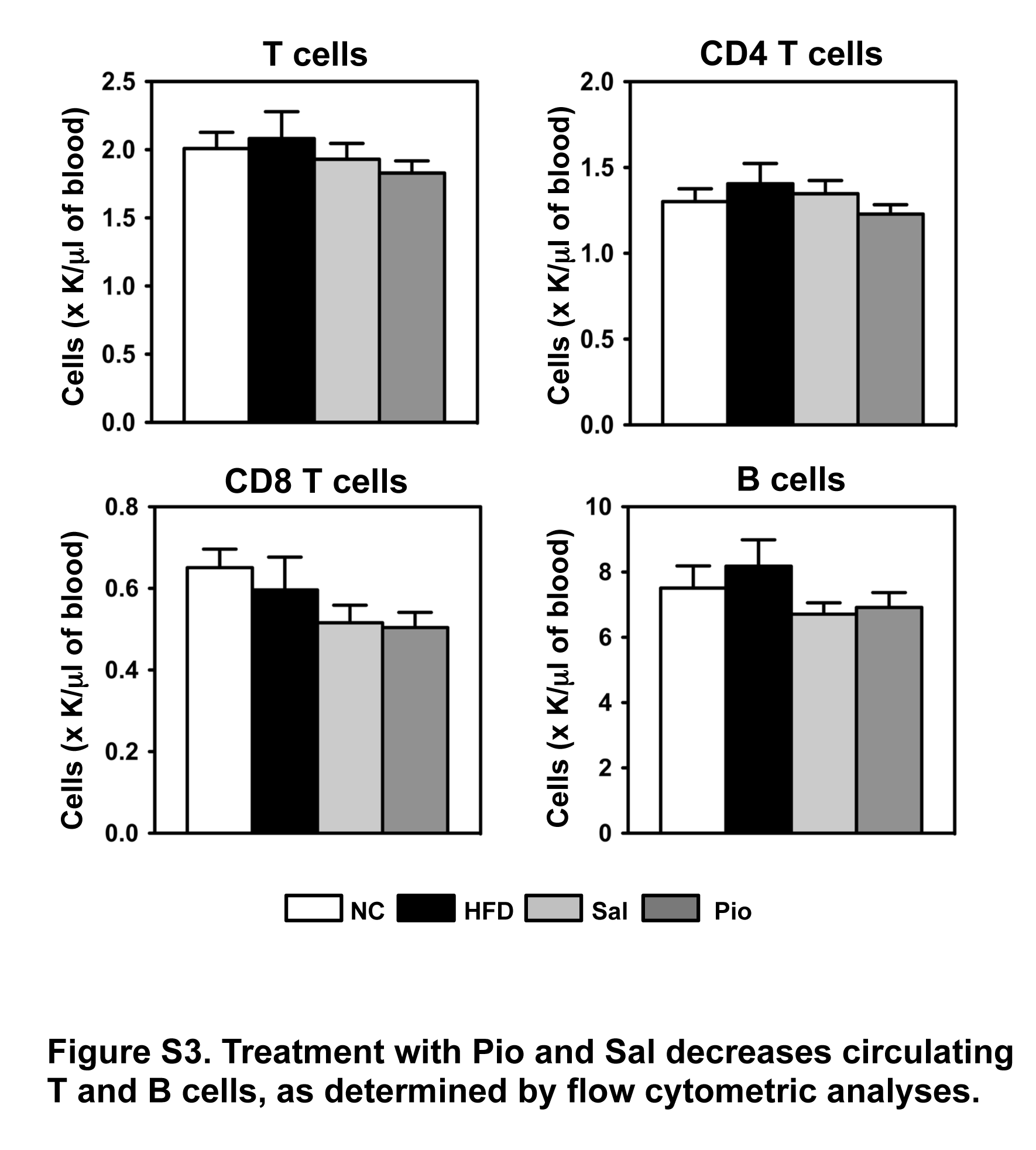

Supplement: Figure S3 — Treatment with Pio and Sal decreases circulating T and B cells, as determined by flow cytometric analyses. Blood was collected from the tail vein in the presence of 5 mM EDTA, incubated with BD Fc Block, stained with antibodies and lysed with FACS Lysing Solution (BD Biosciences). The cells were then analyzed with LSR II and the data were analyzed by FlowJo software. (TIF) [file pone.0082847.s003.tif]

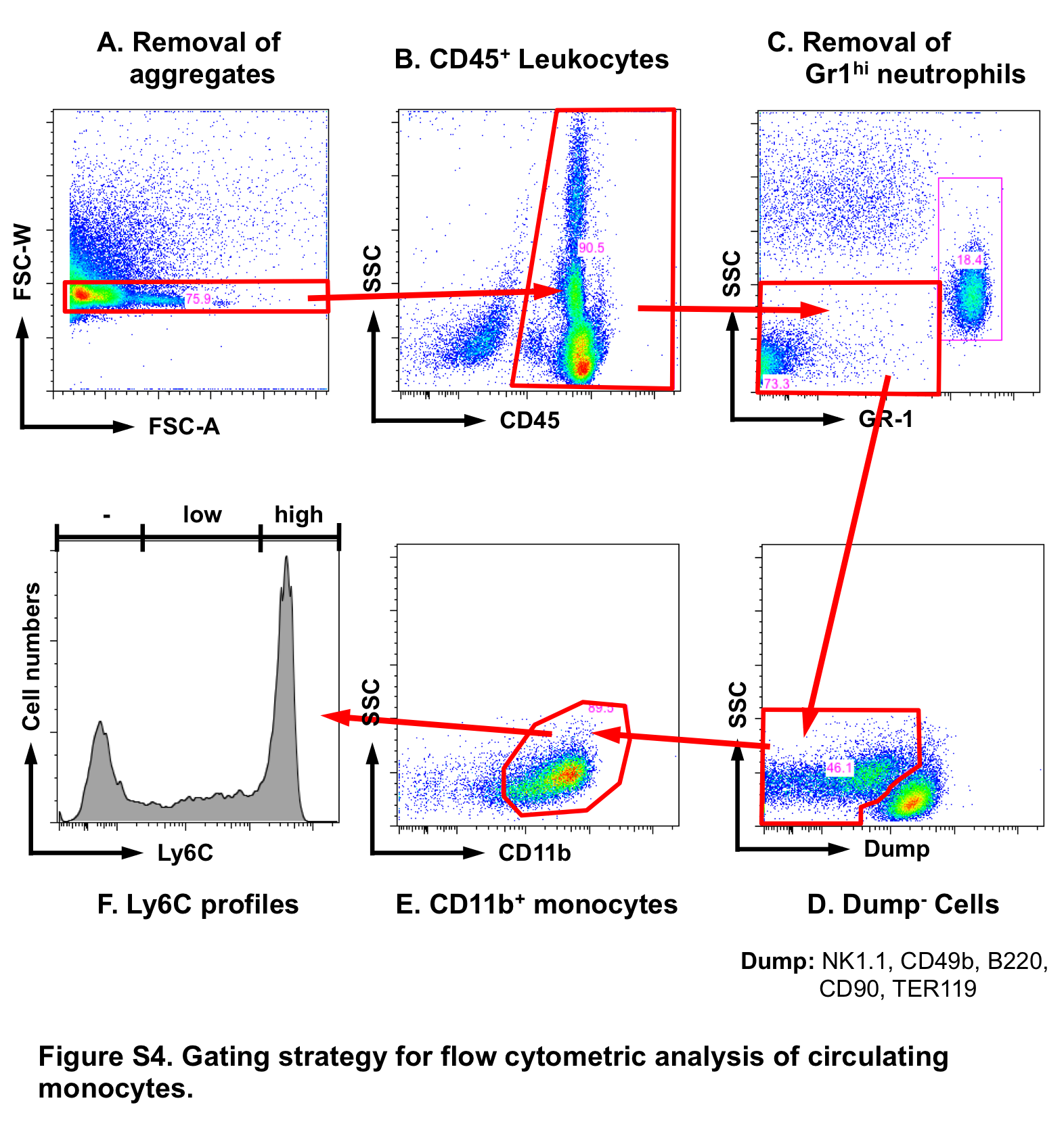

Supplement: Figure S4 — Gating strategy for flow cytometric analysis of circulating monocytes. Whole blood was incubated with FcBlock to block the non-specific binding of Abs and then stained with antibodies against cell-specific surface markers. After staining, the RBCs were removed by lysing with FACS Lysing Solution (BD Biosciences). The cells were then analyzed with LSR II. The data were analyzed by FlowJo software with the following gating strategy. Aggregated cells were first removed by FSC-W/SSC gating (A). The total leukocytes were identified by positivity for CD45 (B). The neutrophils were removed on the basis of their Gr-1hi status (C). The lymphocytes and RBCs were excluded by removing the NK1.1+ (NK and NKT cells), CD49b+ (NK cells), B220+ (B cells), CD90+ (T cells), and TER119+ (RBC) cells (D). Monocytes were selected on the basis of their CD11b positivity (E). The monocyte subpopulations were determined on the basis of their Ly6C expression levels (F). (TIF) [file pone.0082847.s004.tif]

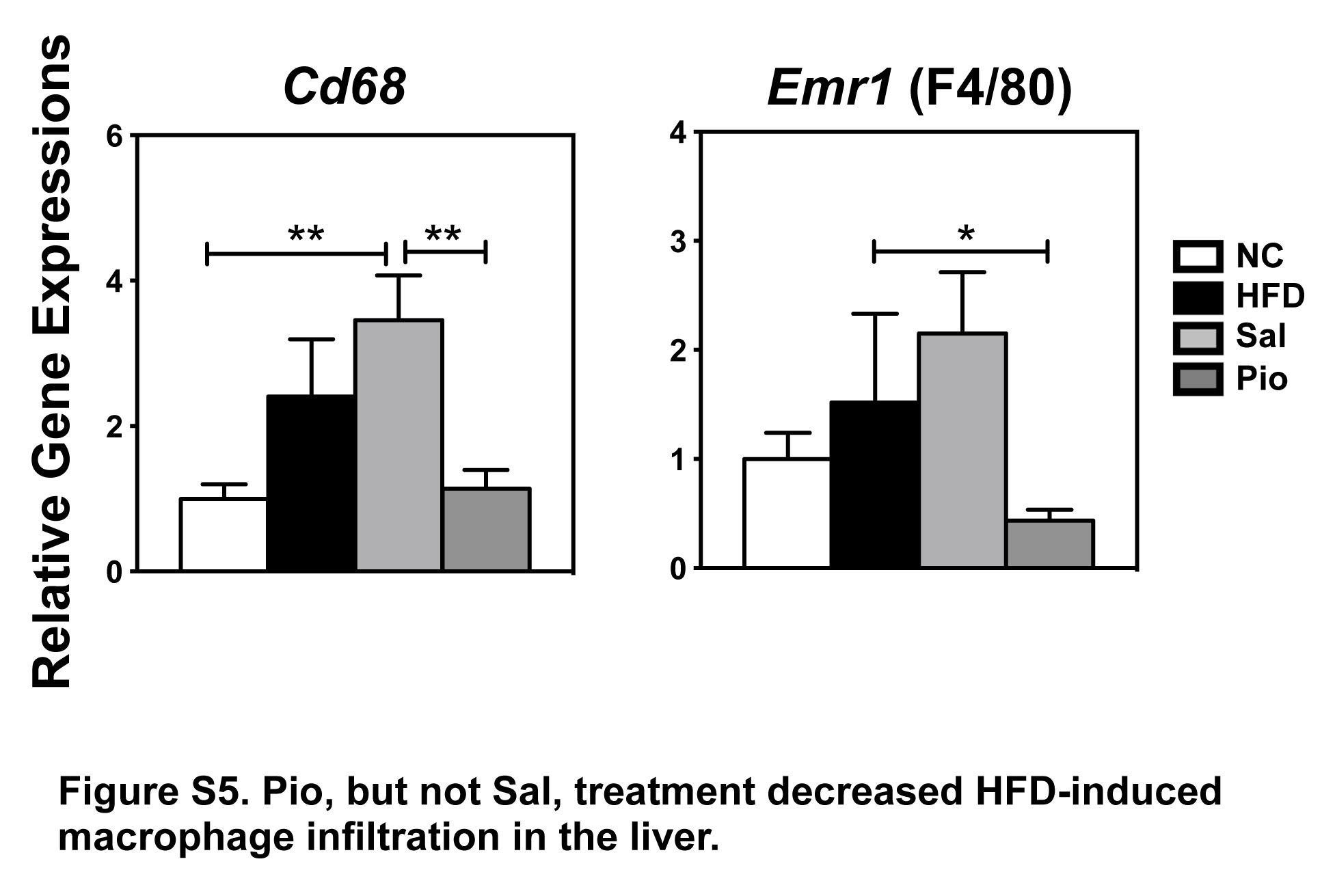

Supplement: Figure S5 — Pio, but not Sal, treatment decreased HFD-induced macrophage infiltration in the liver. Liver samples from the treatment study described in Figure 1 were prepared (n = 6–8 mice per group). mRNA was purified and the expression levels of macrophage-specific genes were determined by real-time RT-PCR. Gene expression was normalized by using the cyclophilin gene. *p<0.05; **p<0.01. (TIF) [file pone.0082847.s005.tif]

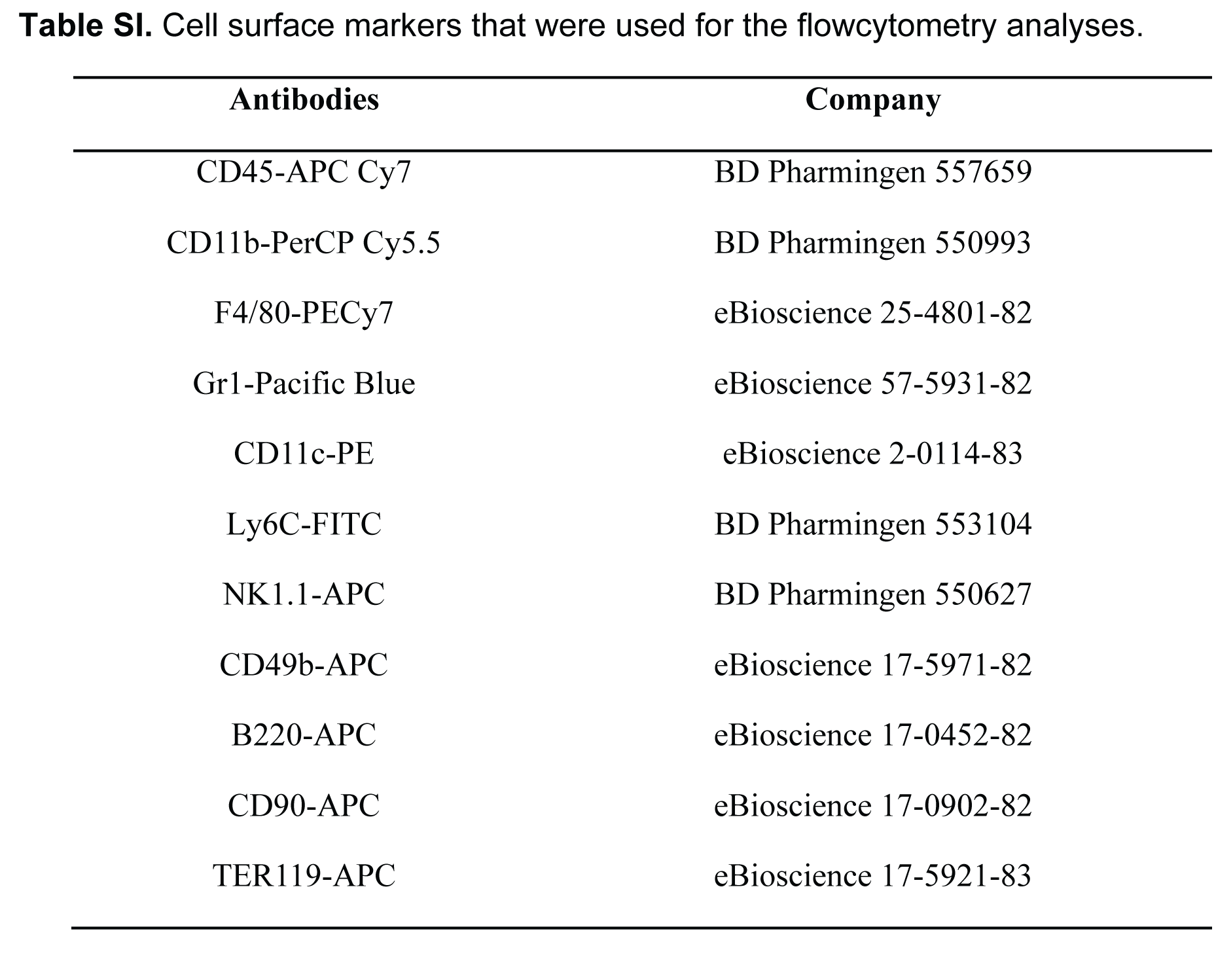

Supplement: Table S1 — Cell surface markers that were used for the flowcytometry analyses. (TIF) [file pone.0082847.s006.tif]

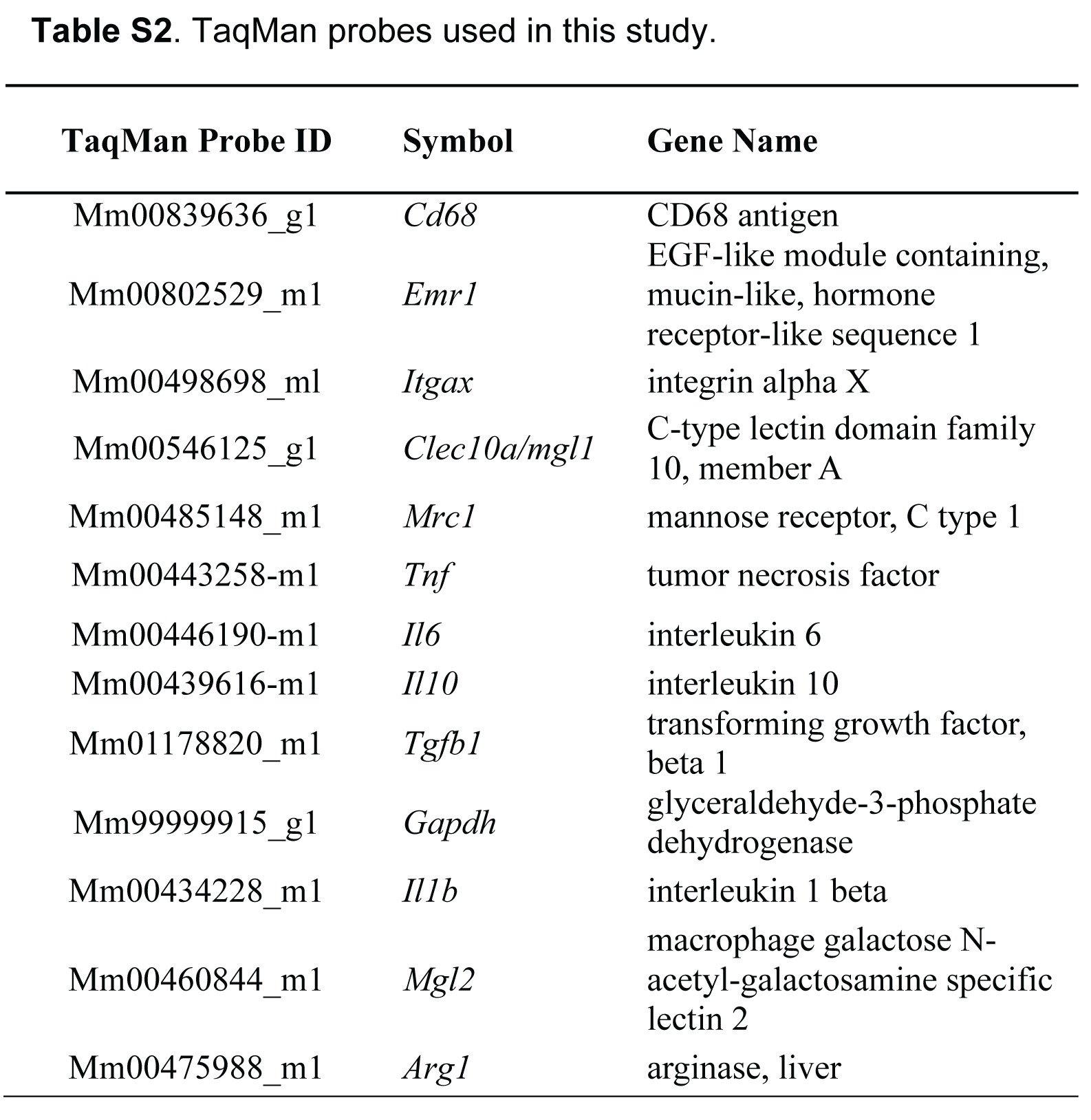

Supplement: Table S2 — TaqMan probes used in this study. (TIF) [file pone.0082847.s007.tif]

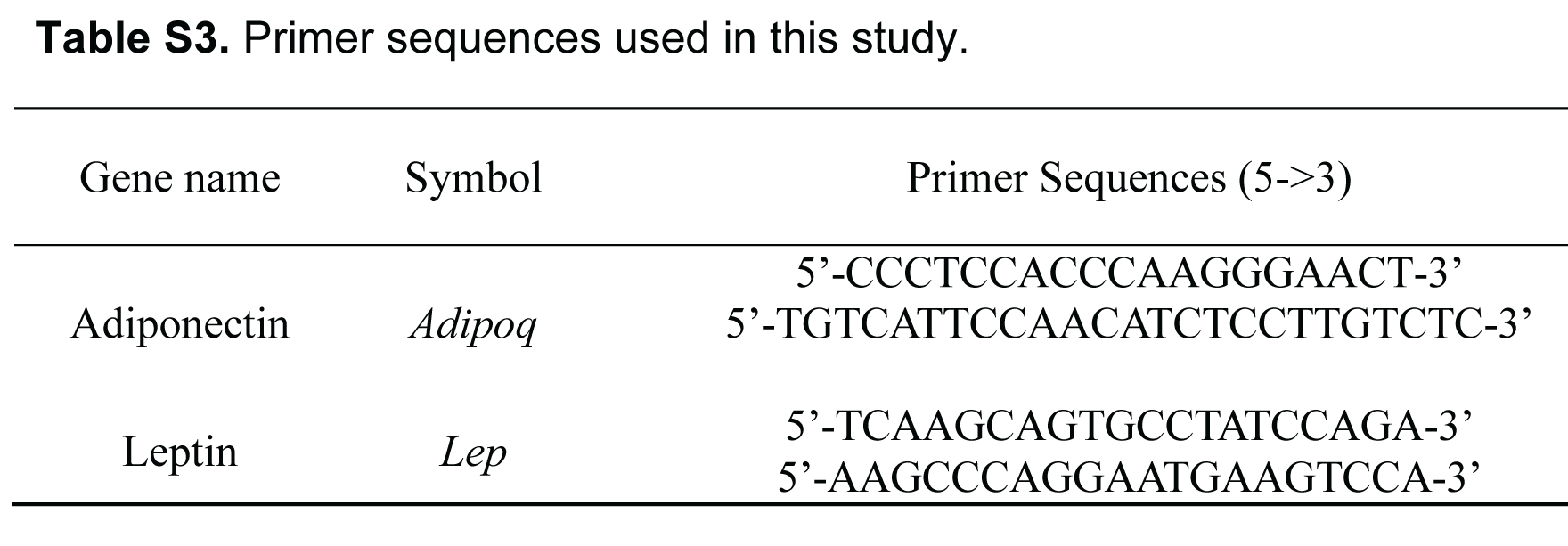

Supplement: Table S3 — Primer sequences used in this study. (TIF) [file pone.0082847.s008.tif]

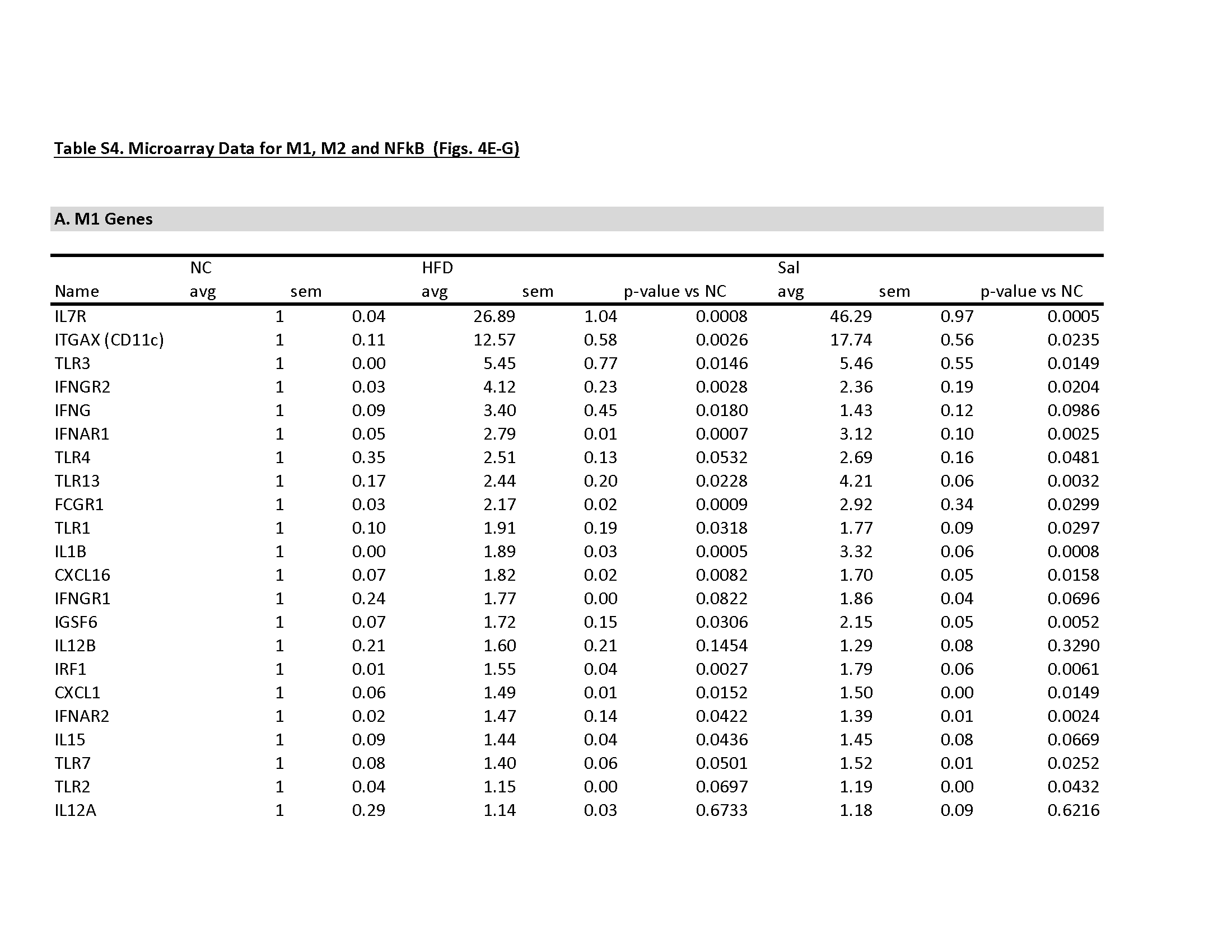

Supplement: Table S4 — (ZIP) [file pone.0082847.s009.zip › Table_S4_Page_1.tiff]

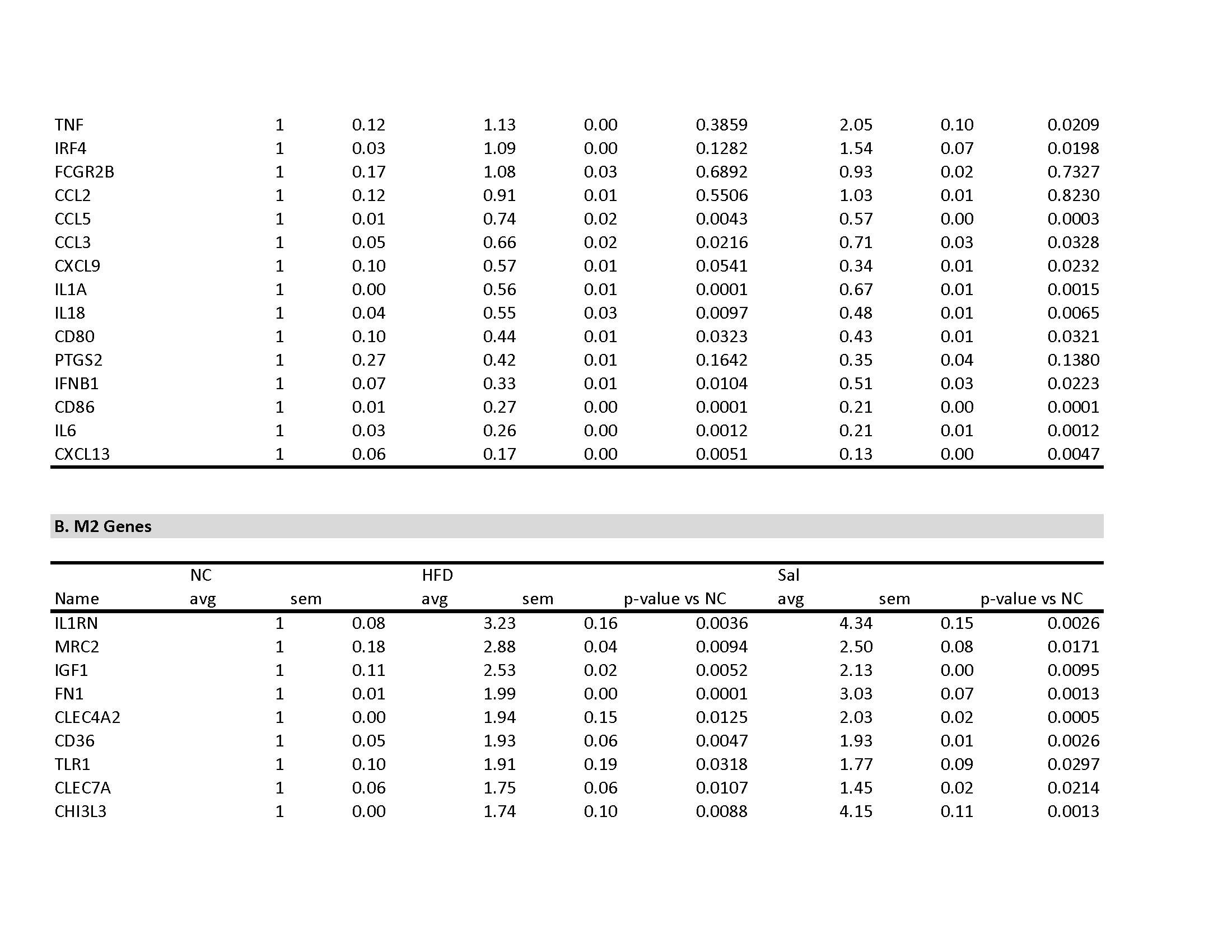

Supplement: Table S4 — (ZIP) [file pone.0082847.s009.zip › Table_S4_Page_2.tiff]

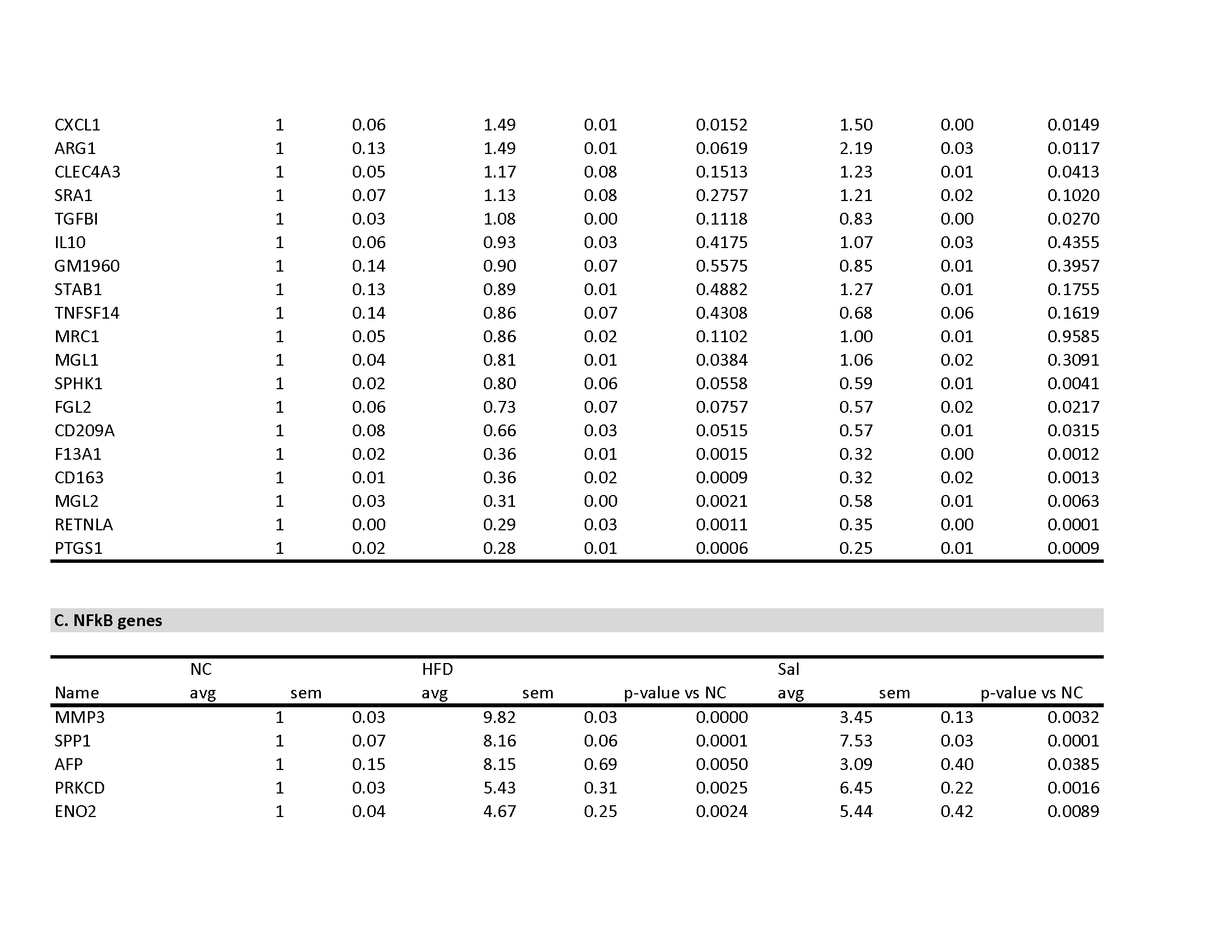

Supplement: Table S4 — (ZIP) [file pone.0082847.s009.zip › Table_S4_Page_3.tiff]
